# Prognostic model construction and validation of esophageal cancer cellular senescence-related genes and correlation with immune infiltration

**DOI:** 10.3389/fsurg.2023.1090700

**Published:** 2023-01-25

**Authors:** Shiyao Zheng, Nan Lin, Qing Wu, Hongxin He, Chunkang Yang

**Affiliations:** ^1^ College of Clinical Medicine for Oncology, Fujian Medical University, Fuzhou, China; ^2^ Department of Gastrointestinal Surgical Oncology, Fujian Provincial Cancer Hospital, Fuzhou, China; ^3^ Fuzong Clinical Medical College of Fujian Medical University, Fujian Medical University, Fuzhou, China; ^4^ Department of Oncology, Molecular Oncology Research Institute, The First Affiliated Hospital of Fujian Medical University, Fuzhou, China

**Keywords:** cellular senescence, esophageal cancer, bioinformatics, immune infiltration, prognosis

## Abstract

**Introduction:**

Cellular senescence is a cellular response to stress, including the activation of oncogenes, and is characterized by irreversible proliferation arrest. Restricted studies have provided a relationship between cellular senescence and immunotherapy for esophageal cancer.

**Methods:**

In the present study, we obtained clinical sample of colon cancer from the TCGA database and cellular senescence-related genes from MSigDB and Genecard datasets. Cellular senescence-related prognostic genes were identified by WGCNA, COX, and lasso regression analysis, and a cellular senescence-related risk score (CSRS) was calculated. We constructed a prognostic model based on CSRS. Validation was performed with an independent cohort that GSE53625. Three scoring systems for immuno-infiltration analysis were performed, namely ssGSEA analysis, ESTIMATE scores and TIDE scores.

**Result:**

Five cellular senescence-related genes, including H3C1, IGFBP1, MT1E, SOX5 and CDHR4 and used to calculate risk score. Multivariate regression analysis using cox regression model showed that cellular senescence-related risk scores (HR=2.440, 95% CI=1.154-5.159, p=0.019) and pathological stage (HR=2.423, 95% CI=1.119-5.249, p=0.025) were associated with overall survival (OS). The nomogram model predicts better clinical benefit than the American Joint Committee on Cancer (AJCC) staging for prognosis of patients with esophageal cancer with a five-year AUC of 0.946. Patients with high CSRS had a poor prognosis (HR=2.93, 95%CI=1.74-4.94, p<0.001). We observed differences in the distribution of CSRS in different pathological staging and therefore performed a subgroup survival analysis finding that assessment of prognosis by CSRS independent of pathological staging. Comprehensive immune infiltration analysis and functional enrichment analysis suggested that patients with high CSRS may develop immunotherapy resistance through mechanisms of deacetylation and methylation.

**Discussion:**

In summary, our study suggested that CSRS is a prognostic risk factor for esophageal cancer. Patients with high CSRS may have worse immunotherapy outcomes.

## Introduction

Esophageal cancer (EC) is the eighth most common cancer-related death worldwide disease (1–3). At present, clinical treatment of EC mainly includes surgery, chemotherapy, radiotherapy, targeted therapy and their combinations (4, 5). Approximately half of the patients have distant metastases when EC is diagnosed, surgery is no longer applicable (6). Unfortunately, radiotherapy, chemotherapy, and targeted therapy have made only limited progress in recent years in improving the generally disappointing outcome (6). Reaching the efficacy benefit of immunotherapy for EC remains challenging.

Cellular senescence (CS) is a stable cell cycle arrest that occurs in diploid cells and limits their proliferative life span, which induces a proliferative arrest in cells at risk of malignant transformation and is therefore widely considered as an anti-tumor mechanism (7, 8). The physiological role of the immune checkpoints is to prevent excessive immune response by termination immune system activation at appropriate time, which can be utilized by tumor to catalyze the auto-destruction of the immune responses (9, 10). Expression of the immune checkpoint PD-L1 was confirmed to be required for senescent cells to evade T-cell immunity, as well as for tumor cells (11).

Cellular senescence-based drugs are currently being explored and developed in two categories, senolytics and senomophics, including senescence-associated secretory phenotype (SASP) inhibitors (12, 13). Immunotherapy involving CS-based drugs seems to be a new therapeutic approach, but the role in the EC remains poorly defined. Thus, we hypothesized that CS-related genes promote EC progression by affecting immune regulation and constructed a prognostic model.

## Materials and methods

### Data acquisition

**Figure 1:**
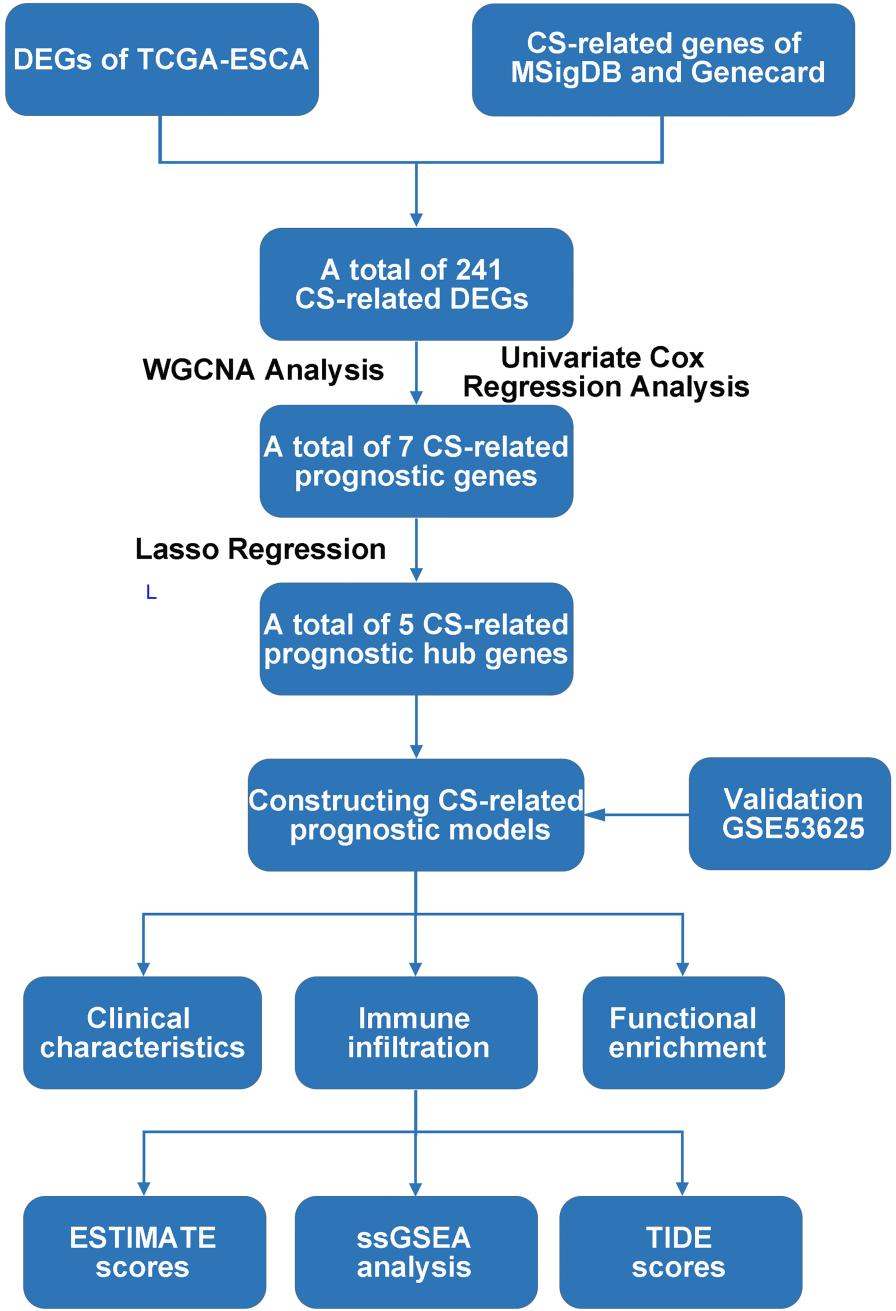
Flow chart of the present study. DEG: Differentially expression gene; TCGA, The Cancer Genome Atlas; CS, Cellular senescence; MSigDB, Molecular signatures database; DEGs, Differentially expressed genes; WGCNA, Weighted correlation network analysis; ESTIMATE, Estimation of STromal and Immune cells in MAlignant Tumours using Expression data; ssGSEA, Single sample gene set enrichment analysis; TIDE, Tumor Immune Dysfunction and Exclusion.

Transcriptomic data and clinical information of esophageal cancer (EC) derived from the TCGA-ESCA cohort as a training set (https://portal.gdc.cancer.gov/), involving 162 EC samples and 11 normal samples. Clinical information not available or ambiguous was removed. Independent cohort GSE53625 as validation set available from GEO database. Cellular senescence-related genes (CSRGs) were selected by the Molecular Signatures Database (MSigDB, http://www.gsea-msigdb.org/) and Genecards (https://www.genecards.org/) tools (Supplementary Table S1). The procedure detailed in this study is shown in Figure 1.

### Identification of CS-related prognostic hub genes

Statistical analyses based on the TCGA database were performed with R. The differentially expressed genes (DEGs) in tumor and normal tissues of TCGA-ESCA cohort were screened by differential analysis. Combined with CS-related genes, CS-related DEGs in EC were initially screened by Venn analysis. The WGCNA weighting analysis of the distribution of correlation modules of these genes was performed, and CS-related prognostic genes were further obtained by univariate COX regression analysis. Finally, CS-related prognostic hub genes were identified by LASSO regression.

### Construction and validation of CS-related risk scores prognostic models

Based on the coefficients of CS-related prognostic hub genes gained from Lasso regression analysis, the CS-related risk scores (CSRS) were constructed as follows.

$$\mathrm{CS}\_\mathrm{related}\;\mathrm{risk}\;\mathrm{scores}(\mathrm{CSRS})=\sum_{i=1}^{n}\mathrm{expressio}n_{\mathrm{gene}\_i}\times \mathrm{lasso}\_\mathrm{coeffieicen}t_{\mathrm{gene}\_i}$$

Independent prognostic factors were screened by univariate and multivariate COX regression analysis. These factors and CSRS were combined to construct a nomogram model for predicting survival in patients with EC. A preliminary assessment was performed with a calibration correction curve.

Data from the GSE53625 dataset was taken to validate the reliability of the model. The effectiveness of the nomogram model was demonstrated by the decision curve analysis (DCA) curve, Kaplan-Meier (KM) curve and receiver operating characteristic (ROC) curve.

### Correlation between CSRS with clinical characteristics and survival

The Wilcoxon signed-rank sum test was used to compare the differences in clinical characteristics of patients in high- and low- CSRS groups. The prognostic value of CSRS for patients of different age groups, pathological staging, and pathological stages was performed by Kaplan-Meier.

### Correlation between CSRS and immune cell infiltration

In the present study, three scoring systems for immuno-infiltration analysis were performed, namely ssGSEA analysis (14), ESTIMATE scores (15) and TIDE scores (16). Levels of infiltration of different immune cells in tumors were quantified by the ssGSEA algorithm through the GSVA package (17). The purity of tumor immune infiltration and abundance of stromal cells were calculated by ESTIMATE algorithm through the estimate package. The dysfunction score and exclusion scores from the TIDE scoring system were applied to predict the efficacy of immunotherapy in different CTL-related subgroups of patients.

### Functional enrichment analysis

GO analysis and KEGG analysis for probing the potential biological functions of gene networks in different modules of the WGCNA with the clusterProfiler package and org.HS.eg.db package (18). The biological mechanisms leading to differences in high and low CSRS groups were explored *via* gene set enrichment analysis (GSEA) by the clusterProfiler package (17, 18).

## Result

### Screening and identification of CS-related prognostic genes

**Figure 2:**
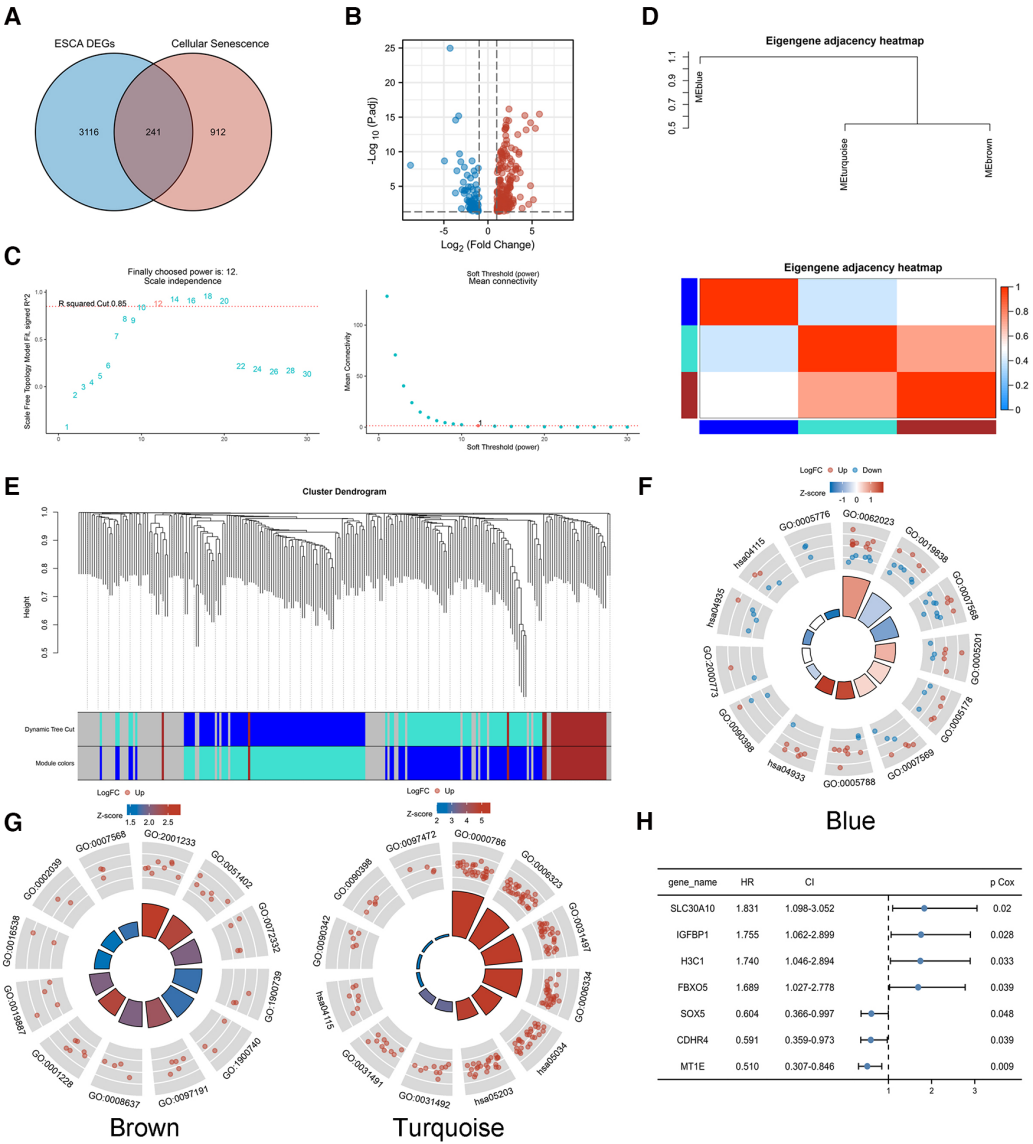
Identification of CS-related prognostic genes. (**A,B**) A total of 241 genes were differentially expressed between EC and normal tissues(|log2FC|>1, *p* < 0.05). (**C**) Soft threshold *β* of WGCNA was determined as 12 based on the scale-free fit and the mean connectivity. (**D–E**) WGCNA network classified the CS-related DEGs into three different modules, blue, brown and turquoise. GO/KEGG analysis was performed in module genes. (**F**) Blue module. (**G**) Brown and turquoise modules. (**H**) Univariate COX regression analysis of modular genes.

A total of 1,153 CS-related genes were derived by MSigDB and Genecards tools (Supplementary Table S1), of which 241 genes (Figure 2A) were differentially expressed between EC and normal tissues (Figure 2B, |log_2_FC|>1, *p* < 0.05).

WGCNA analysis of TCGA-ESCA transcriptome data was performed to search for highly related gene modules. Based on the relationship between the soft threshold with the scale-free fit and the mean connectivity, a suitable soft threshold *β* was finally determined as 12 (Figure 2C). The network classified the CS-related DEGs into three different modules, blue, brown and turquoise (Figures 2D,E), by using a dynamic tree cutting and clustering algorithm. The correlation between modules was presented by a heat map, which showed that the turquoise module was highly genetically correlated with the brown module.

GO/KEGG analysis was performed to probe the biological functions associated with each module gene. The genes of the blue module were mainly enriched in cellular senescence and aging (Figure 2F). The genes of the brown and turquoise modules (Figure 2G) might play a role in biological processes such as cellular senescence, as well as, apoptosis-related signaling pathways. The detailed GO/KEGG annotations are presented in Table 1.

**Table 1:** GO/KEGG analysis annotations of module genes.

ONTOLOGY	ID	Description
BP	GO:2000773	negative regulation of cellular senescence
BP	GO:0090398	cellular senescence
BP	GO:0007568	aging
BP	GO:0007569	cell aging
BP	GO:0007568	aging
BP	GO:2001233	regulation of apoptotic signaling pathway
BP	GO:1900739	regulation of protein insertion into mitochondrial membrane involved in apoptotic signaling pathway
BP	GO:1900740	positive regulation of protein insertion into mitochondrial membrane involved in apoptotic signaling pathway
BP	GO:0072332	intrinsic apoptotic signaling pathway by p53 class mediator
BP	GO:0051402	neuron apoptotic process
BP	GO:0008637	apoptotic mitochondrial changes
BP	GO:0097191	extrinsic apoptotic signaling pathway
BP	GO:0006323	DNA packaging
BP	GO:0031497	chromatin assembly
BP	GO:0006334	nucleosome assembly
BP	GO:0090342	regulation of cell aging
BP	GO:0090398	cellular senescence
CC	GO:0005776	autophagosome
CC	GO:0062023	collagen-containing extracellular matrix
CC	GO:0005788	endoplasmic reticulum lumen
CC	GO:0000786	nucleosome
MF	GO:0005178	integrin binding
MF	GO:0005201	extracellular matrix structural constituent
MF	GO:0019838	growth factor binding
MF	GO:0019887	protein kinase regulator activity
MF	GO:0002039	p53 binding
MF	GO:0001228	DNA-binding transcription activator activity, RNA polymerase II-specific
MF	GO:0016538	cyclin-dependent protein serine/threonine kinase regulator activity
MF	GO:0031492	nucleosomal DNA binding
MF	GO:0031491	nucleosome binding
MF	GO:0097472	cyclin-dependent protein kinase activity
KEGG	hsa04115	p53 signaling pathway
KEGG	hsa04935	growth hormone synthesis, secretion and action
KEGG	hsa04933	AGE-RAGE signaling pathway in diabetic complications
KEGG	hsa04115	p53 signaling pathway
KEGG	hsa05034	Alcoholism
KEGG	hsa05203	viral carcinogenesis

Univariate COX regression analysis of the modular genes identified seven genes that were strongly associated with overall survival (OS), namely SLC30A10, IGFBP1, H3C1, FBXO5, SOX5, CDHR4 and MT1E (Figure 2H). The above genes were identified as CS-related prognostic genes.

### Development of CS-related risk scoring system and construction as well as validation of CSRS nomogram model

**Figure 3:**
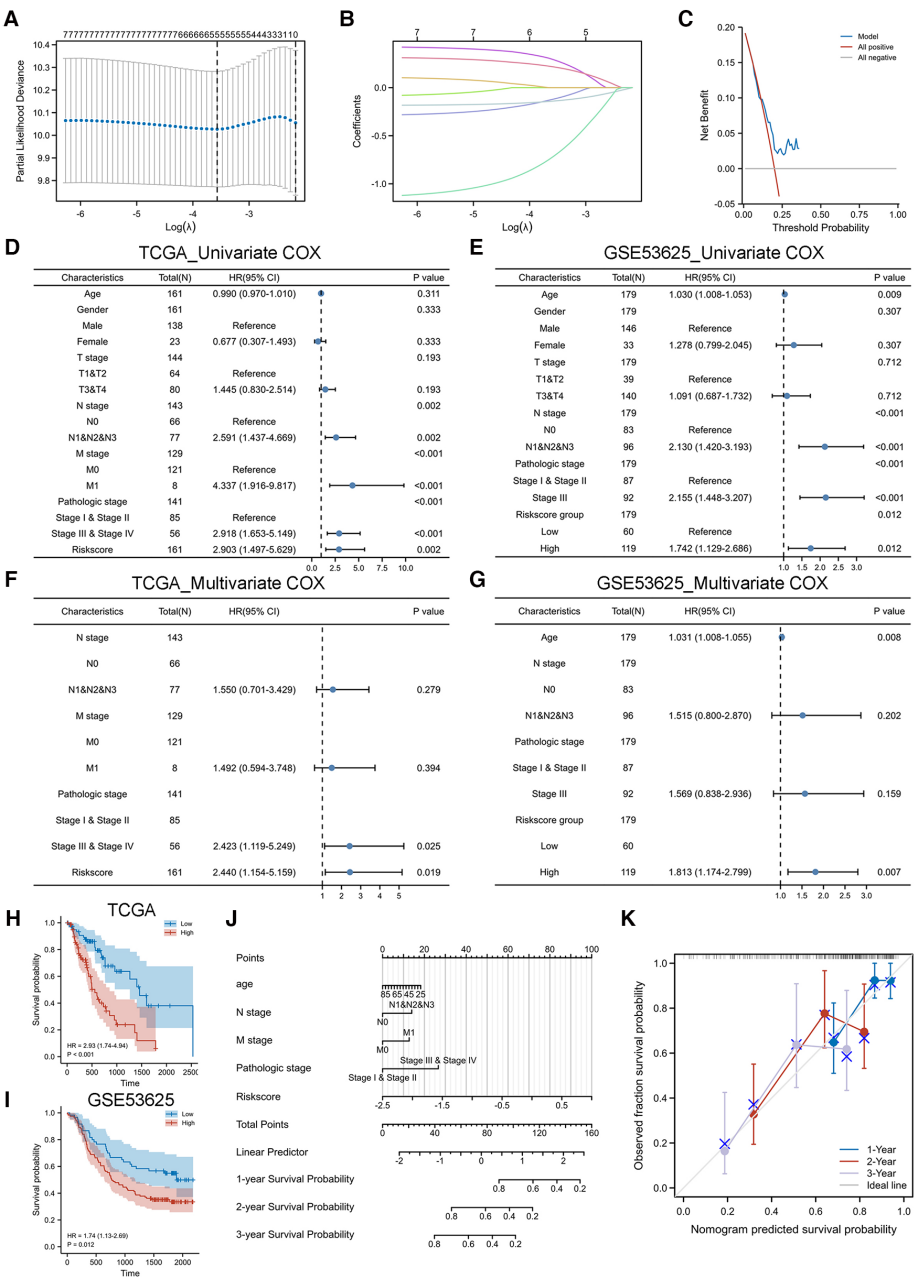
Construction and validation of CSRS nomogram model. (**A,B**) Five genes were identified as CS-related prognostic hub genes by lasso algorithm, including IGFBP1, H3C1, SOX5, CDHR4 and MT1E. (**C**) DCA chart confirmed the prognostic utility of CSRS. (**D-G**) Univariate and multivariate Cox regression analyses of OS in TCGA-ESCA. Validation is performed by GSE53625. (**H-I**) KM curves of OS in TCGA-ESCA and GSE53625. (**J**) Nomogram model to predict the 1-,2- and 3-year survival of EC patients. (**K**) Calibration curves for evaluating. The fit is around the diagonal and the C-index value is 0.744, indicating good consistency of the model.

The regression coefficients (Table 2) of the above 7 CS-related prognostic genes were calculated by the Lasso algorithm (Figures 3A,B) using OS as an outcome indicator, with the

CSRS=0.2901×H3C1+0.2158∗IGFBP1−0.7121∗CDHR4−0.1390∗MT1E−0.1184∗SOX5

The prognostic DCA chart (Figure 3C) confirmed the utility of the CSRS scoring system in predicting survival outcomes in patients with EC.

**Table 2:** The regression coefficients 7 CS-related prognostic genes.

Gene id	Coefficients
H3C1	0.29014853
IGFBP1	0.21577076
SLC30A10	0
FBXO5	0
SOX5	−0.11840431
MT1E	−0.1390272
CDHR4	−0.71213391

We performed a COX regression analysis of the TCGA-ESCA cohort to uncover factors affecting the prognosis of esophageal patients. In the independent cohort GSE53625, EC patients were divided into high-risk and low-risk groups based on the median CSRS in TCGA-ESCA as the cutoff value for further analysis to verify the generalizability of the CSRS score. The results of the univariate COX analysis in the TCGA cohort (Figure 3D) suggested that N stage, M stage, pathological stage and CSRS (HR = 2.903, 95%CI = 1.497–5.629, *p* = 0.002) were risk factors affecting the prognosis of esophageal cancer, which was similarly validated in the GSE53625 cohort (Figure 3E, risks score group: HR = 1.742, 95%CI = 1.129–2.686, *p* = 0.012). Further multivariate COX analysis at TCGA-ESCA (Figure 3F) and GSE53625 (Figure 3G) indicated the reliability of the prediction of prognosis in patients with EC by CSRS. CSRS can accurately distinguish esophageal cancer patients with different survival times, which means that a higher CSRS represents a worse prognosis as reflected by the results of the KM analysis (Figures 3H,I).

Integrating the above analysis, we constructed a nomogram model to predict the 1-,2- and 3-year survival of EC patients based on N stage, M stage, pathological stage and CSRS (Figure 3J). The fit is around the diagonal and the C-index value is 0.744, indicating good consistency of the model (Figure 3K). In addition, we evaluated the efficacy of the nomogram model. The DCA curve (Supplementary Figure S1A) results showed that the prediction of survival outcome in patients with EC using the CSRS was superior to that using American Joint Committee on Cancer (AJCC) staging. The benefit of prediction using our constructed nomogram model was greater than that of CSRS and AJCC. The KM curve (Supplementary Figure S1B) results showed that patients with high nomogram scores had a worse prognosis (HR = 5.35, 95% CI = 2.61–10.96, *p* < 0.001). The accuracy of the nomogram model in predicting the 1-(AUC = 0.781),3-(AUC = 0.754) and 5 (AUC = 0.946) years’ prognosis of patients with EC was also assessed by time-dependent ROC analysis (Supplementary Figure S1C).

### Clinicopathological characteristics and prognostic value in different CSRS groups

**Figure 4:**
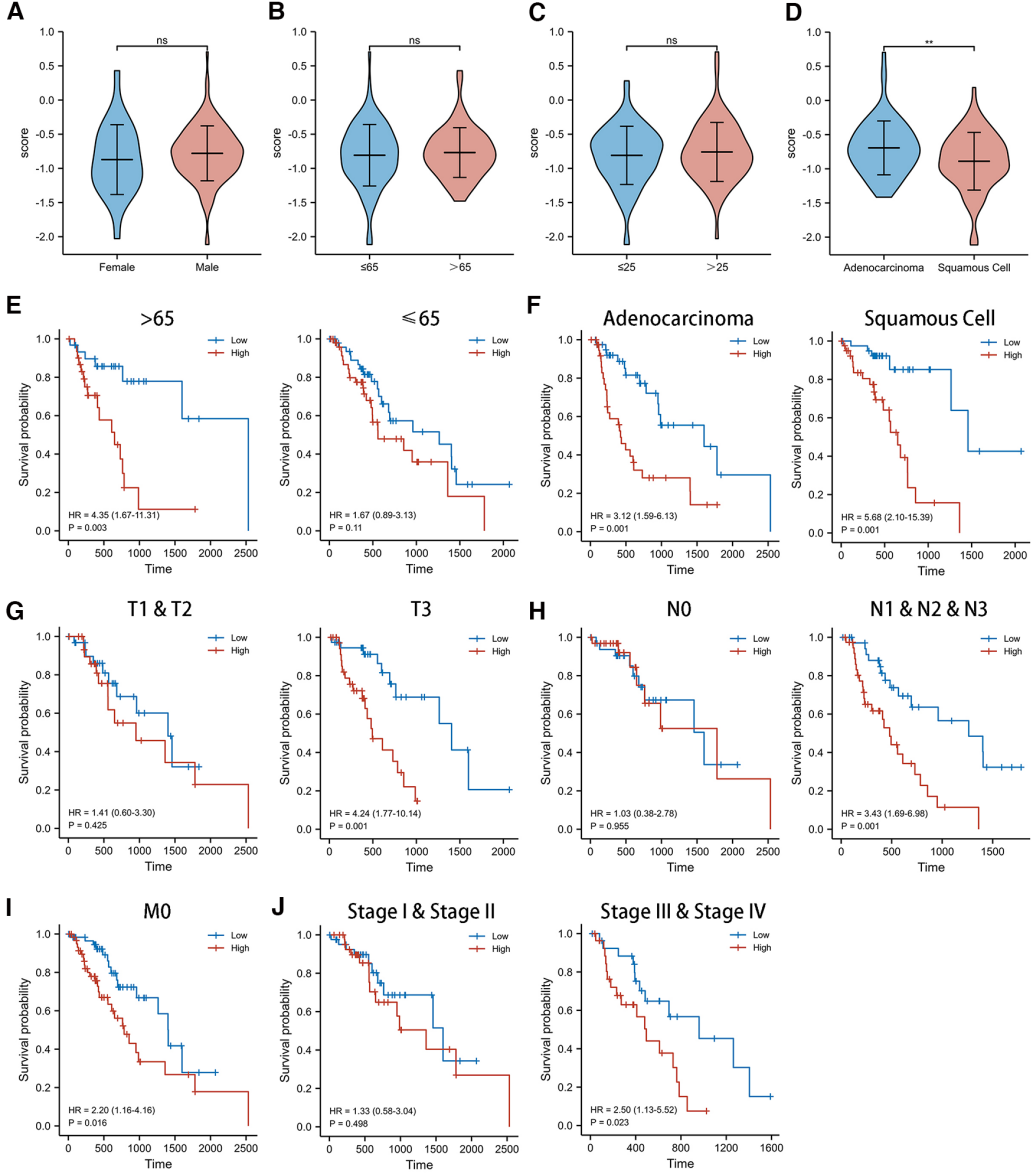
Clinicopathological characteristics and survival analysis of different CSRS groups. (**A,B**) CSRS distribution showed no significant differences in different gender (**A**), age (**B**) and BMI (**C**), while CSRS was higher in patients with EAC than ESCC (**D**). Subgroup survival analysis of age (**E**), pathological staging (**F**), T stage (**G**), N stage (**H**), M stage (**I**) and pathological stage (**J**) between high- and low-CSRS patients. EAC, Esophageal adenocarcinoma; ESCC, Esophageal squamous cell cancer; ns: No significance; **p < 0.01.

We observed no significant difference in the distribution of CSRS among EC groups by gender (Figure 4A), age (Figure 4B), and BMI (Figure 4C). However, in terms of pathological type (Figure 4D), CSRS was higher in patients with esophageal adenocarcinoma (EAC) than those with esophageal squamous cell cancer (ESCC).

For this reason, we investigated the prognostic value of CSRS in different subgroups of patients with EC (Figure 4E–J). CSRS accurately determined prognosis in patients with either EAC (HR = 3.12, 95%CI = 1.59–6.13, *p* = 0.001) or ESCC (HR = 5.68, 95%CI = 2.10–15.39, *p* = 0.001), as well as in patients with EC aged more than 65 years (HR = 4.35, 95%CI = 1.67–11.31, *p* = 0.003) or T3 stage (HR = 4.24, 95%CI = 1.77–10.14, *p* = 0.001) or N1&N2&N3 stage (HR = 3.34, 95%CI = 1.69–6.98, *p* = 0.001) or M0 stage (HR = 2.20, 95%CI = 1.16–4.16, *p* = 0.016) or pathological stage III & IV (HR = 2.50, 95%CI = 1.13–5.52, *p* = 0.023). However, for patients aged less than 65 years, T1 & T2 stages, N0 stages, and pathological stages I & II, CSRS scores were not good predictors of prognostic outcome.

### Multidimensional immune infiltration analysis in different CSRS groups

**Figure 5:**
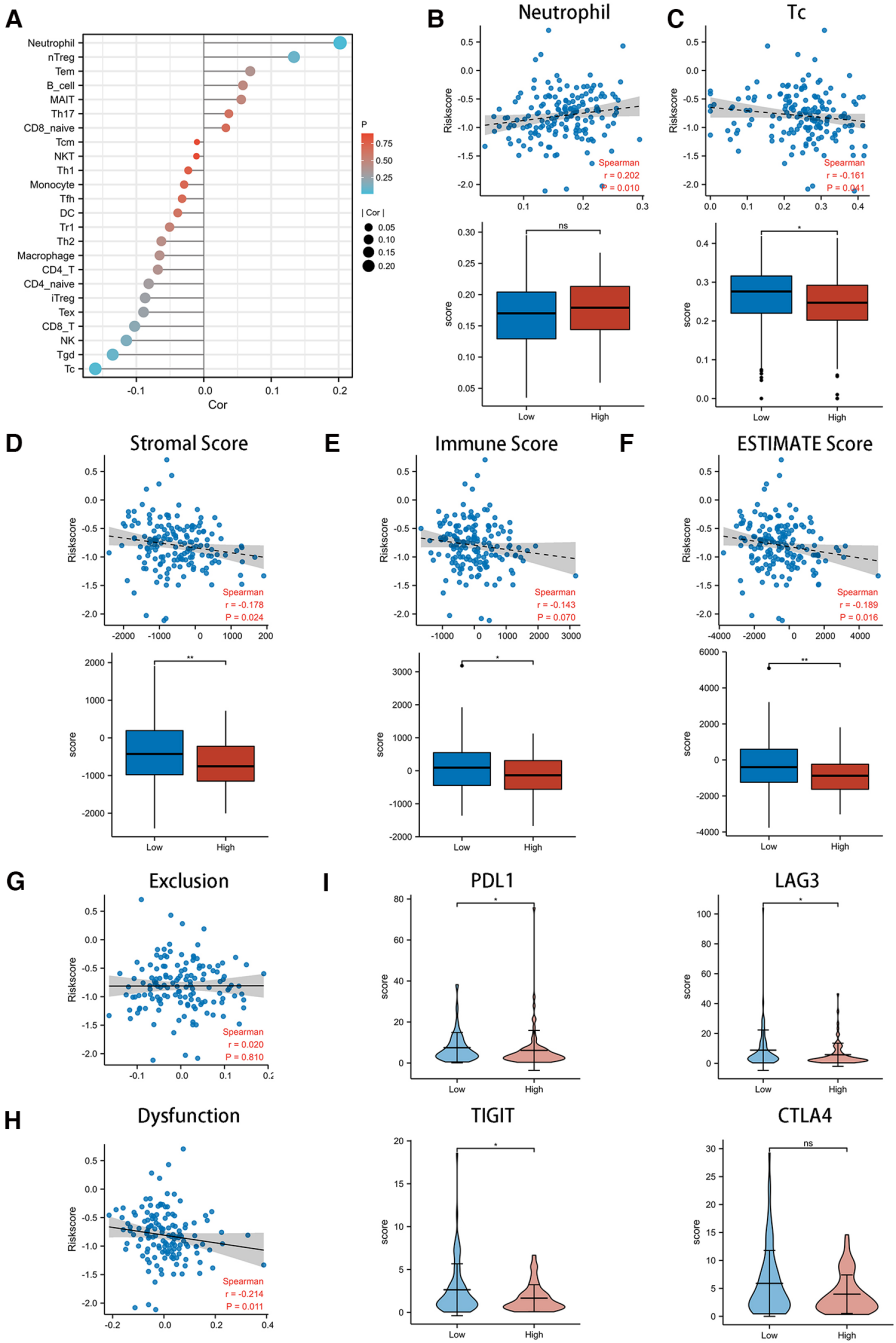
Exploring the role of CSRS in the immunotherapy of esophageal cancer. (**A**) Correlation of CSRS with immune cell infiltration was performed by ssGSEA analysis. High CSRS group were infiltrated by more neutrophil (**B**) and fewer Tc(**C**). Relationship between scores with CSRS, as well as comparison of scores between high- and low-CSRS group in stromal (**D**) score, immune (**E**) score and ESTIMATE (**F**) score. Relationship between exclusion (**G**) and dysfunction (**H**) scores with CSRS. (**I**) Comparison of checkpoint genes, including PDL1, LAG3, TIGIT and CTLA4, between high- and low-CSRS groups. ns: no significance; *: *p* < 0.05; **: *p* < 0.01.

We adopted three scoring systems to analyze tumor immune infiltration in EC patients with different CSRS groups, namely ssGSEA analysis (Figures 5A–C), ESTIMATE score (Figures 5D–F) and TIDE score (Figures 5G,H). EC patients in the high CSRS group were infiltrated by fewer Tc and Tgd cells, while there was a positive correlation with the infiltration of neutrophil cells. Stromal scores (*r* = −0.178, *p* = 0.024) and ESTIMATE scores (*r* = −0.189, *p* = 0.016) were observed to be negatively correlated with CSRS, whereas not immune scores. There were differences in all three scores between the high- and low-CSRS groups of esophageal cancer. The TIDE scoring system is commonly used to evaluate the efficacy of immunotherapy in oncology patients, including the exclusion score and dysfunction score of T cells. CSRS was negatively correlated with dysfunction scores (*r* = −0.214, *p* = 0.011), and no significant correlation was observed with exclusion scores. We subsequently compared the expression of immune checkpoint-related genes in different CSRS groups (Figure 5I). High expression of PDL1, LAG3 and TIGIT were observed in low-CSRS group (*p* < 0.05) than high-CSRS group.

### Potential biological mechanisms in different CSRS groups

**Figure 6:**
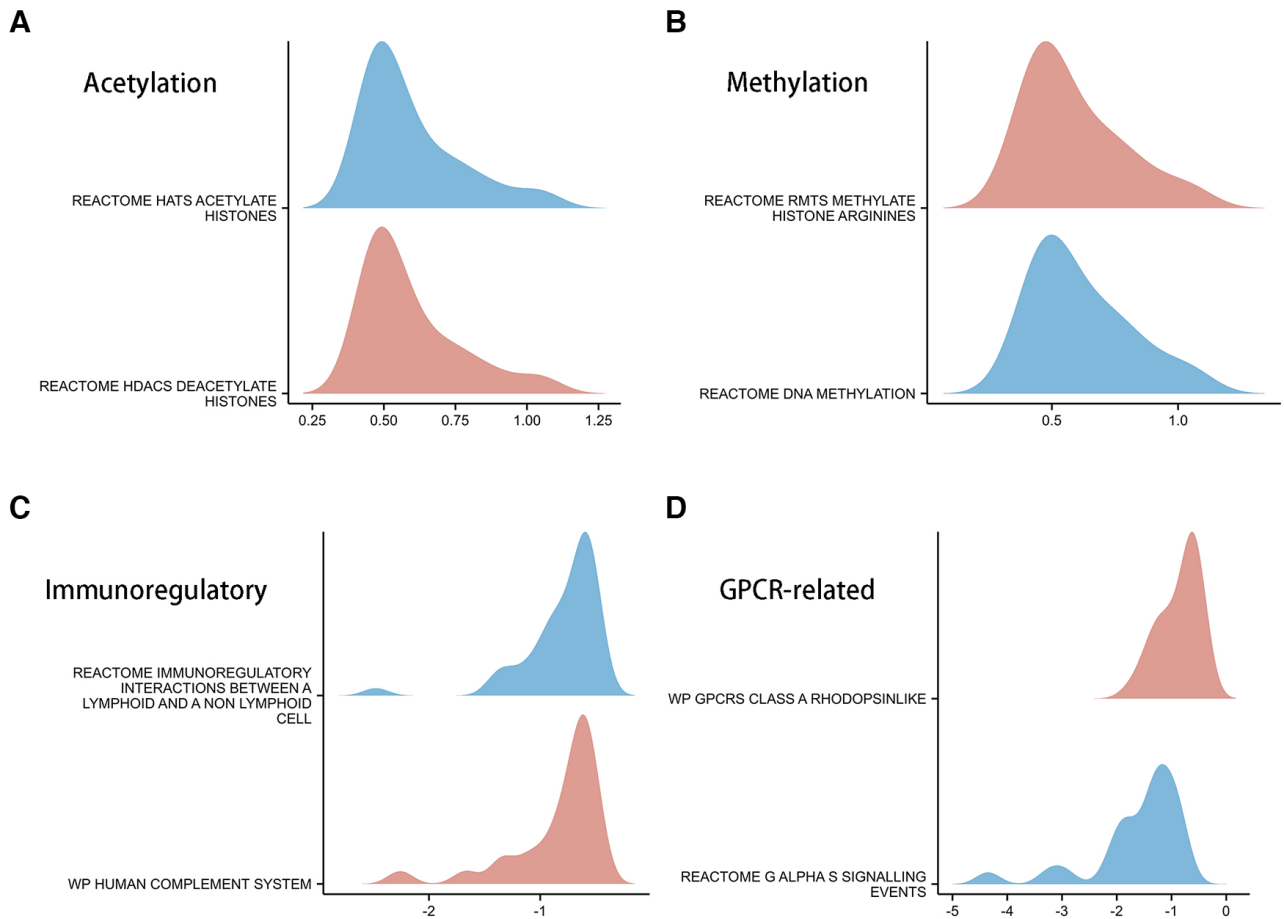
GSEA analysis in high- and low-CSRS group. (**A**) Acetylation-related pathways, including HATs acetylate histones and HDACs deacetylate histones. (**B**) Methylation-related pathways, including RMTs methylate histone arginine and DNA methylation. (**C**) Immunomodulatory-related pathways, including immunoregulatory interactions between a lymphoid and a non-lymphoid cell and human complement system. (**D**) GPCR-related pathways, including GPCRs class A rhodopsinlike and G*α*S signalling events.

In order to explore the biological mechanisms leading to differences between high- and low- CSRS groups, GSEA analysis was performed. The results showed that the high-CSRS group was positively enriched in acetylation- (Figure 6A) and methylation-related (Figure 6B) pathways, and negatively enriched in immunomodulatory (Figure 6C) and GPCR-related pathways (Figure 6D).

## Discussion

Cellular senescence (CS) is a cellular response to stress, including the activation of oncogenes, characterized by irreversible proliferation arrest (8). Cellular senescence was first discovered and described by Hayflick and Moorhead (19). They found that human cell cultured *in vitro* lost their ability to proliferate and entered a state of growth arrest after 50 to 70 generations of continuous culture. In recent years, as cellular senescence has been studied more intensively, DNA damage response, endoplasmic reticulum stress and induction of antiapoptotic genes have been defined as the phenotypes of cellular senescence (20–24).

Some reports have suggested that the microenvironment of CS is associated with cancer progression, such as the SASP (25–27). SASP mediates chronic inflammation and stimulates the growth of cancer, while SASP also enhances cell cycle arrest, prompting immune cells to defend cancer (28, 29). There were limited studies on CS and esophageal cancer(EC), whereas identification of CS-related genes with clinical significance is crucial for immunotherapy studies of EC. Thus, we hypothesized that CS-related genes promote EC progression by affecting immune regulation.

In the present study, 241 CS-related DEGs were initially screened from TCGA-ESCA. The WGCNA network classified the CS-related DEGs into three different modules which were associated with the CS and apoptosis pathways. We finally identified five CS-associated prognostic genes in EC by COX analysis and the Lasso regression algorithm, including H3C1, IGFBP1, MT1E, SOX5 and CDHR4.

H3C1 is a member of histone family (30). Missense mutations in histone related genes promote tumor progression, a process known as oncohistones, which is a major challenge for tumor treatment (31, 32). Yi.H et al. revealed for the first time that high expression of histone deacetylase 7 (HDAC7) was closely associated with poor in EC, suggesting that HDAC7 is a potential cancer-promoting agent (33). IGFBP1 binds to insulin-like growth factors (IGFs) I and II in plasma, prolonging their half-life period (34). Elevated levels of IGF-1 and IGF-2 are related to various cancers (35–37), including EC (38, 39). The insulin-like growth factor (IGF) signaling pathway plays a key role in cell growth, differentiation, and apoptosis (38). IGFBP1 was identified as a promising biomarker for the diagnosis of early-stage esophageal cancer in a clinical study involving 2028 patients with esophageal cancer at three medical centers (40). However, there have been few biological studies on IGFBP-1 in esophageal cancer. CDHR4, which has been less studied, is a member of the cadherin related family. While cadherin, a key molecule for tumor entry into blood vessels and lymph, is associated with tumor infiltration and metastasis by mediating EMT (41, 42).Our study suggested that high expression of H3C1 and IGFBP1 predicted poor prognosis, while CDHR4 was a prognostic protective factor (Figure 1H), consistent with the results of the currently published studies. SOX5, a member of the SOX (SRY-related HMG-box) family involved in the determination of the cell fate. In a mouse model, SOX5 inhibits glioma formation by inducing acute cellular senescence (43). MT1E is an isoform of MT1, and it has been reported that MT1E expression is positively correlated with esophageal cancer malignancy (44).

We constructed a prognostic model based on CSRS by combining N stage, M stage, and pathological stage, which was validated well in an independent cohort (Figure 3). The DCA curve, KM curve and ROC curve demonstrated the validity of the nomogram model (Supplementary Figure S1). The nomogram model predicts better clinical benefit than AJCC staging for the prognosis of patients with esophageal cancer with a five-year AUC of 0.946. We observed differences in the distribution of CSRS in ESCC and EAC (Figure 4D). Therefore, further subgroup survival analysis was performed (Figures 4E–J). ESCC caused by smoking and alcohol consumption varies from the pathogenesis of EAC by Barrett's esophagus progression (45, 46). According to our analysis, the CSRS score to determine prognosis was not limited by pathological staging. However, CSRS was less effective in judging early-stage EC groups, as well as in younger subgroups. Regarding this observation, we believed that more clinical samples needed to be included for subsequent evaluation.

Immunotherapy has made brilliant achievements in the field of advanced EC treatment, rewriting the treatment paradigm of EC (47, 48). KEYNOTE-590 is the first global multicenter phase III clinical trial exploring the efficacy of immune combination chemotherapy in advanced EC (49). CheckMate −577 provides new high-level evidence for immunotherapy of locally advanced EC (50). We conducted an analysis between CSRS and tumor immune infiltration in EC to investigate whether CSRS contributes to the immunotherapy of EC (Figure 5). Results revealed that the high CSRS group had poor immunotherapy efficacy, while the low CSRS group may have better immunotherapy efficacy based on assessment of immune cell infiltration status, tumor microenvironment, T cell dysfunction and immune checkpoint-related genes.

To further validate the above findings, a GSEA analysis of DEGs in the high- and low- CSRS groups was performed (Figure 6A). The results showed that genes in the high CSRS group were positively enriched in acetylation and methylation related pathways. Negative enrichment was observed on immunomodulatory-related pathways. HDAC promotes tumorigenesis through biological mechanisms such as induction of cell proliferation and inhibition of apoptosis (51–53). Combining HDCA inhibitors with immunotherapy drugs for tumors significantly reverses immunotherapy resistance (54). Abnormal DNA methylation allows highly mutated tumors to evade immune responses through a rapid division mechanism, which is an important factor in tumor resistance to immune responses (55). The above analysis provides direction for higher immunotherapy benefit in patients with high CSRS, and further biological experimental validation will be needed further.

There are still some limitations to our study. Although CSRS was applied to different pathological types of esophageal cancer, it is generally effective in determining the prognosis of patients with early-stage esophageal cancer based on the current data. We believed that this may be due to the bias caused by the small number of cases of TCGA-ESCA, for example, there were only 16 patients with pathological stage I. Subsequently, we will expand the sample size or combine the data from our center to verify the generalizability of CSRS.

## Conclusion

In the present study, we constructed a CS-related prognostic model for EC. Comprehensive analysis, combined with preliminary validation of independent cohort, suggested that CSRS is a prognostic risk factor for EC. Patients with high CSRS may have worse immunotherapy outcomes.

## Data Availability

The original contributions presented in the study are included in the article/**Supplementary Material**, further inquiries can be directed to the corresponding author/s.
