# Supplementary figures and images for: Prognostic model construction and validation of esophageal cancer cellular senescence-related genes and correlation with immune infiltration
Source: Front Surg. 2023 Jan 25;10:1090700. doi: 10.3389/fsurg.2023.1090700 (PMC9905418; doi:10.3389/fsurg.2023.1090700)

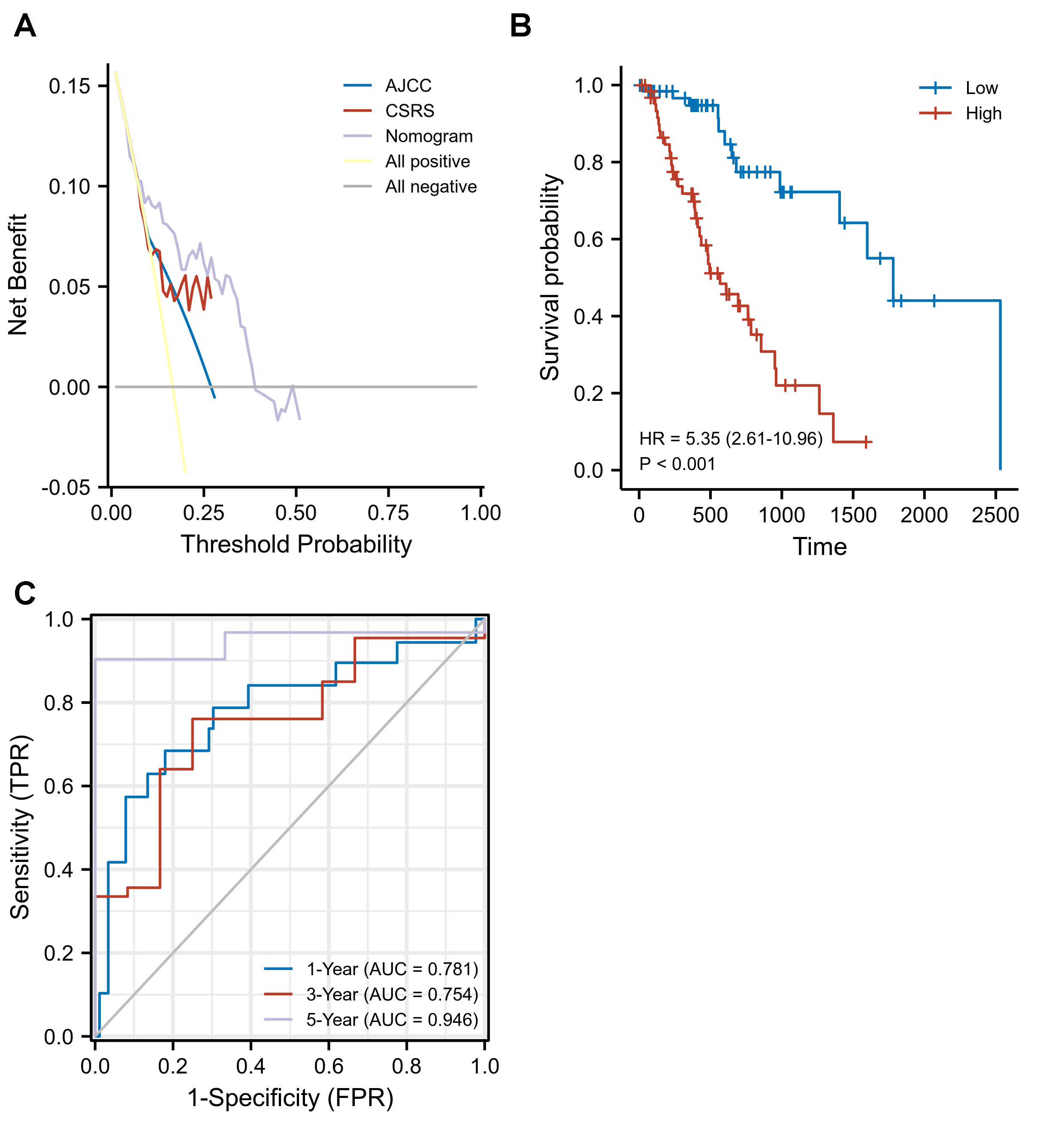

Supplement: Supplementary file 2 [file Image1.jpeg]
